# Quantification of LV function and mass by cardiovascular magnetic resonance: multi-center variability and consensus contours

**DOI:** 10.1186/s12968-015-0170-9

**Published:** 2015-07-28

**Authors:** Avan Suinesiaputra, David A. Bluemke, Brett R. Cowan, Matthias G. Friedrich, Christopher M. Kramer, Raymond Kwong, Sven Plein, Jeanette Schulz-Menger, Jos J. M. Westenberg, Alistair A. Young, Eike Nagel

**Affiliations:** Department of Anatomy with Radiology, University of Auckland, Auckland, New Zealand; National Institute of Biomedical Imaging and Bioengineering, Bethesda, MD USA; Departments of Medicine and Diagnostic Radiology, McGill University, Montreal, Canada; University of Virginia, Charlottesville, VA USA; Harvard Medical School, Boston, USA; University of Leeds, Leeds, UK; Leiden University Medical Center, Leiden, Netherlands; Charité University Medicine Berlin and HELIOS Klinikum Berlin-Buch, Berlin, Germany; Institute for Experimental and Translational Cardiovascular Imaging, DZHK Centre for Cardiovascular Imaging, University Hospital Frankfurt/Main, Frankfurt, Germany

**Keywords:** Left ventricular function, Benchmarks, Reproducibility, Multi-center, Left ventricular mass, Image analysis

## Abstract

**Background:**

High reproducibility of LV mass and volume measurement from cine cardiovascular magnetic resonance (CMR) has been shown within single centers. However, the extent to which contours may vary from center to center, due to different training protocols, is unknown. We aimed to quantify sources of variation between many centers, and provide a multi-center consensus ground truth dataset for benchmarking automated processing tools and facilitating training for new readers in CMR analysis.

**Methods:**

Seven independent expert readers, representing seven experienced CMR core laboratories, analyzed fifteen cine CMR data sets in accordance with their standard operating protocols and SCMR guidelines. Consensus contours were generated for each image according to a statistical optimization scheme that maximized contour placement agreement between readers.

**Results:**

Reader-consensus agreement was better than inter-reader agreement (end-diastolic volume 14.7 ml vs 15.2–28.4 ml; end-systolic volume 13.2 ml vs 14.0–21.5 ml; LV mass 17.5 g vs 20.2–34.5 g; ejection fraction 4.2 % vs 4.6–7.5 %). Compared with consensus contours, readers were very consistent (small variability across cases within each reader), but bias varied between readers due to differences in contouring protocols at each center. Although larger contour differences were found at the apex and base, the main effect on volume was due to small but consistent differences in the position of the contours in all regions of the LV.

**Conclusions:**

A multi-center consensus dataset was established for the purposes of benchmarking and training. Achieving consensus on contour drawing protocol between centers before analysis, or bias correction after analysis, is required when collating multi-center results.

## Background

Left ventricular (LV) mass and volumes are essential for the management of patients with cardiovascular disease. In particular, LV mass (LVM) is an independent predictor of cardiovascular events [1], and end-diastolic volume (EDV) and end-systolic volume (ESV) are associated with adverse remodeling [2]. Cardiovascular magnetic resonance (CMR) is currently the most accurate and reproducible method for quantifying LV mass and volumes [3]. CMR is non-invasive, does not require geometrical assumptions, is non-ionising and has high signal-to-noise ratio. Because of these advantages, CMR is becoming widely used for the measurement of ventricular volumes, function and mass in many clinical centers, as well as in large research studies including the Multi-Ethnic Study of Atherosclerosis (MESA) [4] and the UK Biobank [5].

LV mass and volume quantification requires accurate delineation of the blood pool and myocardium. Although the contrast between flowing blood and the myocardium in steady-state free precession (SSFP) images is typically excellent, the precise placement of the contours is reader dependent [6]. High reproducibility of LV mass and volume measurement based on cine CMR has been shown within single centers [7, 8], but differences in training and standard operating procedures may occur between centers [6]. A multi-center consensus ground truth dataset would be valuable for evaluating sources of variability, training new readers, establishing standard protocols for multi-centre studies and validating new computer algorithms for automated contouring. Such a dataset is difficult to establish, due to the time-consuming nature of manual contouring. Although there are several recourses that offer large breadth [5, 9, 10], no resource offers depth of expert analysis from multiple centers. Greater depth of readers is valuable in evaluating sources of variation and an unbiased consensus. The aim of this study was to develop a consensus ground truth LV contour dataset for SSFP cine images, derived from expert readers representing seven independent CMR centers from many countries around the world, in accordance with the SCMR post-processing guidelines [11].

## Methods

### Participants

Cine CMR images from 15 subjects (five healthy volunteers, six patients with myocardial infarction, two patients with heart failure, and two patients with LV hypertrophy) were included in this study. CMR images were acquired with contiguous short axis slices and 2–3 long axis slices in accordance with SCMR guidelines using three different scanners (4 GE, 5 Siemens and 6 Philips). Spatial resolution varied with FOV, ranging from 92 × 72 to 280 × 280 mm^2^ (see Table 1). Temporal resolution was typically 20–30 frames, except for one case with 60 cardiac frames. Slice thickness was either 8 or 10 mm. Short-axes view series covering the LV from apex to base were defined in 10–15 slices. Anonymized images were contributed to the Cardiac Atlas Project database with the approval of local institutional review boards. Written informed consent was obtained from all participants.

**Table 1:** Patient and image characteristics, showing variability in patient pathologies, gender, age, MR vendors and MR parameters. All units (slice thickness (SLT), slice distance (SLD), field of view (FOV) and pixel size) are in mm. Number of slices and frames are taken from short-axis series

Case	Pathology	Gender	Age	Slices	Frames	SLT	SLD	FOV	Pixel size	Vendor
1	Healthy	M	70	10	20	10	10	205 × 205	1.25 × 1.25	GE
2	Infarct	M	50	13	25	6	10	92 × 75	2.08 × 2.08	GE
3	Heart Failure	F	77	15	20	8	10	211 × 211	1.21 × 1.21	GE
4	Infarct	M	70	12	27	6	10	182 × 137	1.41 × 1.41	SIEMENS
5	Heart Failure	F	63	14	20	8	8	187 × 187	1.37 × 1.37	GE
6	Hypertrophy	M	61	12	20	10	10	187 × 187	1.37 × 1.37	GE
7	Infarct	M	59	11	25	6	10	102 × 102	1.88 × 1.88	SIEMENS
8	Infarct	F	42	11	24	6	10	182 × 142	1.41 × 1.41	GE
9	Infarct	M	52	11	22	6	10	182 × 137	1.41 × 1.41	GE
10	Healthy	M	67	15	30	8	8	208 × 208	1.23 × 1.23	GE
11	Healthy	F	66	11	60	10	10	177 × 177	1.45 × 1.45	SIEMENS
12	Healthy	M	50	13	30	10	10	156 × 156	1.44 × 1.44	SIEMENS
13	Infarct	M	70	15	30	8	8	280 × 280	1.14 × 1.14	SIEMENS
14	Healthy	M	57	15	30	8	8	277 × 277	1.21 × 1.21	PHILIPS
15	Hypertrophy	M	71	15	30	8	8	241 × 241	1.19 × 1.19	PHILIPS

### Analysis

Seven expert readers, representing seven CMR core laboratories from six countries around the world (two USA, Canada, two UK, Germany and Netherlands) analyzed all cases independently. The contours were reviewed by the core laboratory principal investigator and represented the standard practice of each core laboratory. Each center was able to use their usual software, with the condition that all contours were placed manually, or manually corrected if initial contours were found automatically. Contouring was performed in accordance with the SCMR guidelines on standardized image interpretation and post-processing of CMR images [11]. Trabeculae and papillary muscles were included in the blood pool and excluded from the LV mass. No attempt was made to train the readers, influence their analysis or achieve consistent results between readers.

Epicardial and endocardial contours were drawn on the short-axis slices at end-diastole (ED) and endocardial contours drawn at end-systole (ES). The ED and ES frames were pre-determined for all readers by the coordinating center, based on smallest area in a mid-ventricular slice. All readers were asked to contour slices covering the whole ventricle from apex to base, but there was no restriction on which slices to include or exclude.

Readers used a range of software packages. Two readers used OsiriX (Pixmeo, Geneva, Switzerland), five readers used QMass (Medis, Leiden, the Netherlands), and two readers used CMR42 (Circle Cardiovascular Imaging Inc., Calgary, Canada). Two readers used two different software packages. Contours were imported from these software packages and pre-processed using Matlab R2010a (Mathworks, Natick, MA, USA). Consensus contours were only generated if most of the readers (i.e. four or more) contoured the slice; otherwise, no consensus contours were produced.

### Consensus contour estimation

Consensus contours were estimated using the Simultaneous Truth and Performance Level Estimation (STAPLE) method [12]. This method calculated unique contours in each slice that maximized the conditional probability of the consensus given the readers’ contours. Briefly, contours from each reader were first converted to binary images (1 for pixels within the contour, 0 for pixels outside the contour). Since the contour resolution was higher than the original images, the binary images were calculated at a resolution 4x higher than the original image. Given an estimate of the consensus contours, the sensitivity of each reader was calculated as the proportion of pixels inside the consensus contours that were also inside the reader contours. Similarly, the reader specificity was calculated as the proportion of pixels outside the consensus contours, which were also outside the reader contours. The STAPLE method uses Expectation-Maximization [13] to calculate the optimal consensus contour, as well as the reader sensitivity and specificity. There are two steps that are performed iteratively until convergence. The first step (Expectation) estimates the consensus probability given the reader contours and current estimates of sensitivity and specificity. The second step (Maximization) updates the sensitivity and specificity of each reader based on this consensus probability. The result is not the same as simple averaging or pixel voting, since specificity and sensitivity behave as weights during the optimization process, which are not assumed to be equal across all readers. Instead, the voting solution is used as the initial estimate to start the iteration. The STAPLE method has been successfully applied in several medical imaging applications, and was recently used to estimate consensus ground truth contours from automated CMR analysis methods [9].

### Cavity volumes and myocardial mass

LV cavity volumes at ED (EDV) and ES (ESV) were computed by slice summation. Two-dimensional cavity areas were multiplied by the inter slice distance to compute a slice volume. The myocardial mass (LVM) was defined at ED by subtracting EDV from epicardial volume and multiplying by 1.05 g/ml.

### Functional assessment

Root mean squared errors (RMSE) in volumes and mass were computed to measure the agreement between each reader and all the other readers. This was defined as

$$ {E}_i(F)=\sqrt{{\displaystyle \sum_{j=1,\kern0.1em j\ne i}^R{\displaystyle \sum_{k=1}^N\frac{{\left({F}_i(k)-{F}_j(k)\right)}^2}{N\left(R-1\right)}}}} $$

where *E*_*i*_ indicates the RMSE for reader *i*, *j* indicates all other readers, *F* indicates either EDV, ESV, LVM or ejection fraction (EF), *R* is the number of readers, *k* indicates the cases, and *N* is the number of cases. A similar RMSE was applied also to the consensus, denoted by *E*_*C*_(*F*), to measure the agreement between the consensus and all readers:

$$ {E}_C(F)=\sqrt{{\displaystyle \sum_{j=1}^R{\displaystyle \sum_{k=1}^N\frac{{\left({F}_C(k)-{F}_i(k)\right)}^2}{NR}}}} $$

Smaller values of *E*_*C*_(*F*) compared to *E*_*i*_(*F*) for all *i* = 1, 2, … , *R* were taken to indicate functionally acceptable consensus contours.

### Visual assessment

An independent reader with over 10 years experience in CMR, who was not affiliated with any of the participating core laboratories, visually assessed the consensus contours by scoring as either acceptable or unacceptable according to whether the contour was clinically plausible.

### Statistics

Bland-Altman analysis was performed to evaluate variation in volumes and mass across all readers relative to the consensus. The limits of agreement were defined at 95 % of confidence interval from the bias. Individual reader bias was quantified by the mean of the differences from the consensus, and reader precision was quantified by the standard deviation of the differences. Volumes and mass estimated from the consensus contours were calculated with the standard error estimated between the readers and the consensus. Statistical analysis was performed using the open source R statistics package (The R Foundation of Statistical Computing Platform, ver. 3.1.1).

## Results

### Visual assessment

**Fig. 1:**
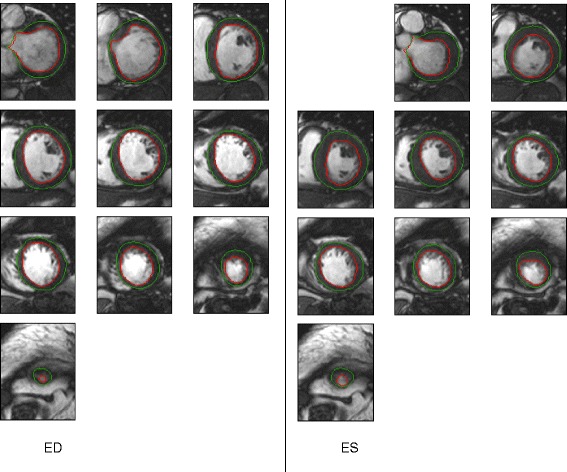
Estimated consensus contours from a myocardial infarction case

**Fig. 2:**
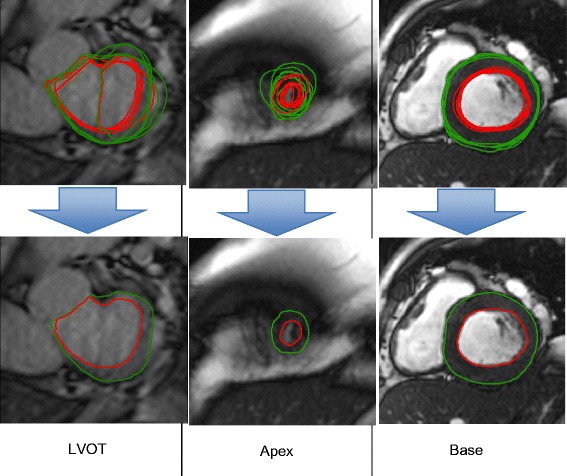
Three examples of difficult cases with larger reader disagreement, showing how STAPLE can estimate consensus contours that gives the best agreement among readers. Top rows: reader contours, bottom rows: the estimated consensus contours

Of all 15 cases, no unacceptable consensus contours were found by the independent reader. Figure 1 shows a representative case of consensus contours estimated from this study. Although large disagreements between readers could be found in some of the apex, base and outflow tract slices, the consensus contours generated from these difficult slices (shown by Fig. 2) were visually acceptable.

### Functional assessment

Table 2 shows the estimated consensus LV function per case in terms of EDV, ESV, LVM and EF (ejection fraction). Standard errors between the readers and the consensus were used to indicate confidence intervals for the estimated values.

**Table 2:** Individual case consensus LV parameters: End-Diastolic Volume in ml (EDV), End-Systolic Volume in ml (ESV), Mass in g (LVM) and Ejection Fraction in % (EF). All values are represented by the consensus estimation ± standard error. Note that LVM was calculated from the ED frame

Case	EDV (ml)	ESV (ml)	LVM (g)	EF (%)
1	104.3 ± 1.8	48.2 ± 2.3	70.4 ± 4.3	53.8 ± 1.5
2	284.9 ± 6.1	186.7 ± 6.7	154.5 ± 10.0	34.5 ± 1.1
3	292.8 ± 4.9	253.5 ± 6.4	134.3 ± 7.4	13.4 ± 1.6
4	190.5 ± 5.0	112.0 ± 4.5	113.7 ± 6.7	41.2 ± 1.2
5	369.0 ± 9.3	267.5 ± 8.2	171.4 ± 11.8	27.5 ± 0.7
6	150.6 ± 4.7	47.7 ± 6.4	174.9 ± 5.8	68.3 ± 3.7
7	190.9 ± 4.8	105.2 ± 4.7	113.5 ± 5.0	44.9 ± 1.2
8	201.3 ± 4.6	141.6 ± 6.9	122.0 ± 5.7	29.7 ± 2.8
9	265.1 ± 7.6	160.7 ± 5.4	130.2 ± 5.1	39.4 ± 0.7
10	157.9 ± 5.6	65.3 ± 4.8	132.7 ± 7.8	58.7 ± 2.0
11	158.9 ± 4.2	69.1 ± 3.1	99.8 ± 5.2	56.5 ± 1.4
12	222.2 ± 5.7	88.3 ± 2.4	121.5 ± 7.3	60.3 ± 1.6
13	216.5 ± 5.3	106.2 ± 5.2	129.1 ± 9.3	50.9 ± 1.5
14	169.7 ± 4.7	74.3 ± 4.7	116.7 ± 6.1	56.2 ± 2.3
15	167.1 ± 5.4	75.8 ± 5.1	193.2 ± 7.3	54.6 ± 2.1

The RMSE values from the consensus (*E*_*C*_) were always the smallest compared to any reader RMSE values (*E*_*i*_). For EDV, *E*_*C*_ was 14.7 ml while *E*_*i*_ was from 15.2 to 28.4 ml. For ESV, *E*_*C*_ was 13.2 ml while *E*_*i*_ was from 14 to 21.5 ml. For LVM, *E*_*C*_ was 17.5 g, while *E*_*i*_ was from 20.2 to 34.5 g. For EF, *E*_*C*_ was 4.2 % while *E*_*i*_ was from 4.6 to 7.5 %.

### Bias and precision

**Fig. 3:**
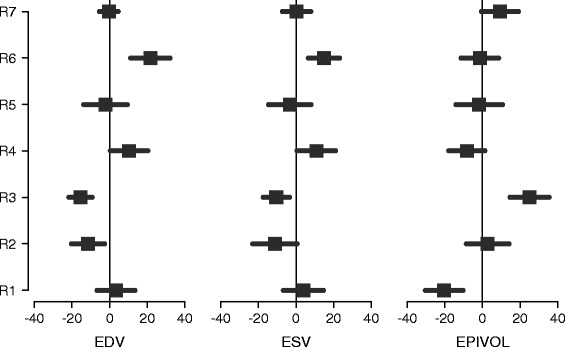
Reader bias and precision against the estimated consensus. Each bar denotes the mean ± one standard deviation

Figure 3 shows reader bias and precision using the estimated consensus values (Table 2) as the reference. All readers showed good precision, with low standard deviations of the differences. The precision in EDV ranged from 5.0 to 11.6 mL (average 8.9 mL); precision in ESV ranged from 7.0 to 11.8 mL (average 9.5 mL); precision in LVM ranged from 10.0 to 12.9 g (average 10.9 g); precision in EF ranged from 1.5 to 5.6 % (average 3.6 %). However, differences in analysis protocols between readers were evident, with some readers exhibiting smaller endocardial and larger epicardial contours, whilst others showed larger endocardial and smaller epicardial contours. The bias in EDV ranged from −36.6 to 40.5 mL (average 0.9 mL); bias in ESV ranged from −32.9 to 41.2 mL (average 0.8 mL); bias in LVM ranged from −44.5 to 59.6 g (average 0.7 g); bias in EF ranged from −11.7 to 12.8 % (average 0.0 %).

**Fig. 4:**
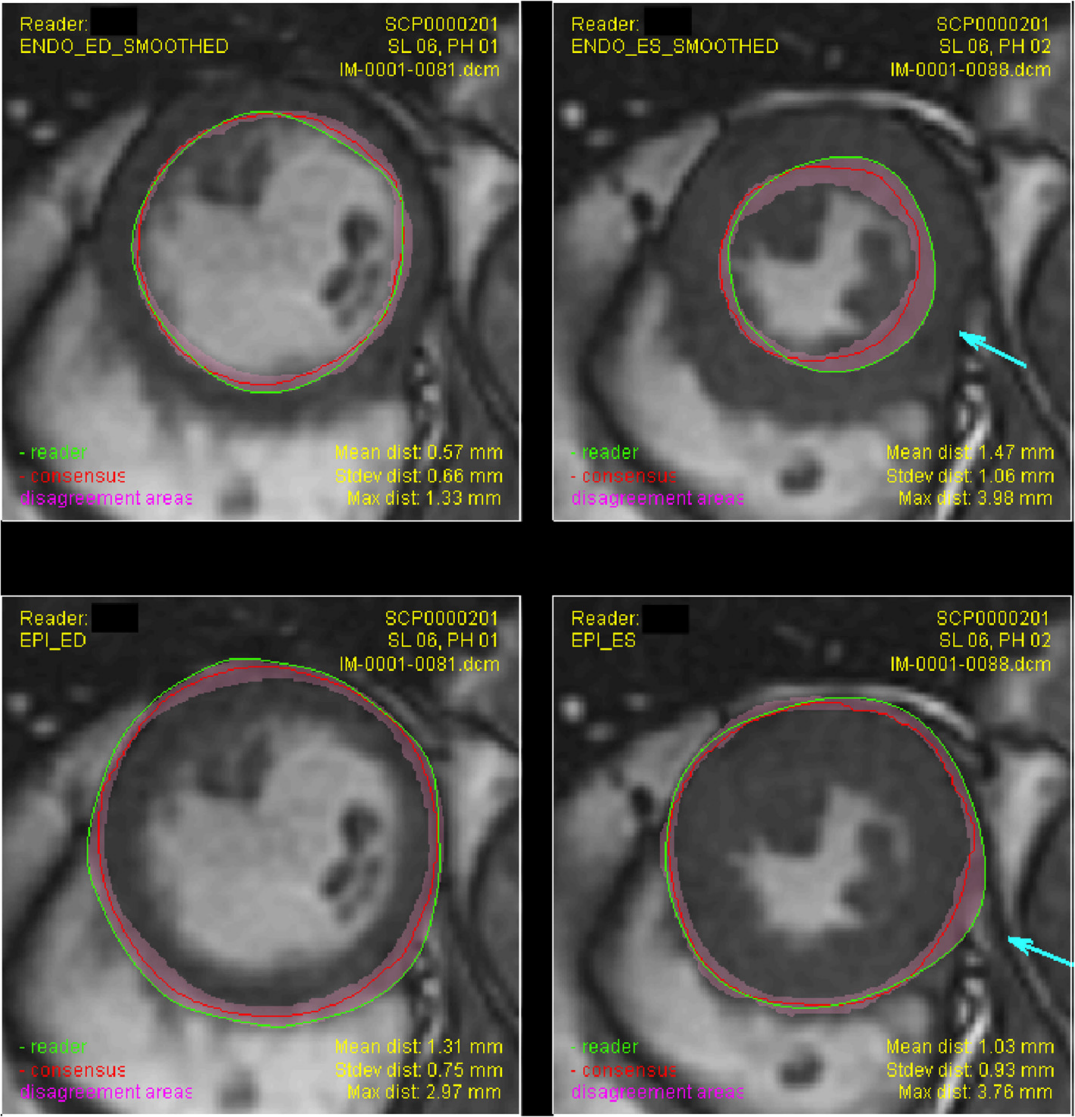
An example of the automatically generated report visualizing the similarity between a reader (green) and consensus (red) contours. Magenta bands indicate the range of contour positions for all seven readers. Arrows indicate points on the reader contour with distance > 3 mm from the consensus contour. Top: endocardial; bottom: epicardial; left: ED; right: ES

Individual reader reports were generated automatically. Figure 4 shows an example report demonstrating similarity of the reader contour with the consensus, with the disparity between expert readers (dark red bands).

## Discussion

Readers were consistent within themselves, which was indicated by the relatively small standard deviations (precision) from the consensus within each reader (Fig. 3). However, different readers could be larger or smaller than the consensus (bias). This was due primarily to differing practices at each core lab, where contouring was consistently smaller or larger compared with the other readers. We also examined other sources of possible bias, in particular in the outflow and apical slices (Fig. 2), but the bias contributions from these slices to the final mass and volume estimate were insignificant.

For clinical studies collating results across core labs, steps should be taken to reduce bias between core labs, either prior to the analysis (by training) or after analysis (by bias correction). This study provides a standard set of cases that could be used as a training set. Alternatively, automated post-hoc bias correction methods [14] can also be applied.

### Consensus contour quality

The consensus LV function estimates had the better agreement with all readers than any individual reader. All the RMSE values for EDV, ESV, mass and EF were smallest for the consensus compared with any reader. This indicated that the consensus had better agreement with the readers than any individual reader.

As demonstrated by Fig. 1, the consensus contours were visually acceptable in all cases. Greatest disagreements between readers appeared in the areas where tissue contrast ratios were low, such as in the apical slices and in the outflow tract. Even for these slices, the estimated consensus contours were visually acceptable. Note that the outflow tract was contoured as part of the LV cavity as recommended in the SCMR guidelines [11].

**Fig. 5:**
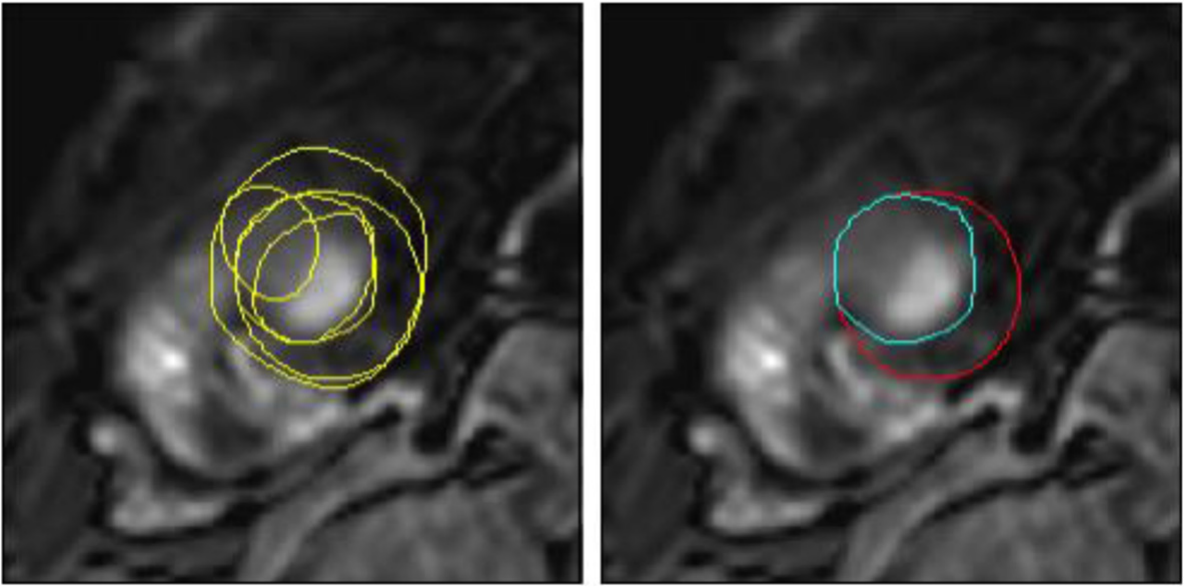
Left: epicardial apical ED contours drawn by readers. Right: the difference of consensus between pixel voting (cyan contour) and the STAPLE algorithm (red contour)

The STAPLE method was performed on each slice and each contour type independently, without taking into account any information about the geometry or anatomy of the myocardium or any pixel intensity values. The STAPLE method is not a vote counting mechanism, and therefore the functional consensus is not a simple average of mass or volume across readers. An example of the difference between voting and STAPLE consensus contours is shown in Fig. 5, demonstrating that STAPLE found an acceptable epicardial apex contour whereas voting did not.

### Reader assessment

In this study, we developed a resource CMR dataset, where myocardial contours were defined through a consensus of seven independent expert readers. The dataset will be useful for training and assessing new readers on contouring CMR images. Feedback to new readers can include graphical displays of areas of maximum disagreement (Fig. 4). Quantitative measurements can be measured in terms of mean, maximum and standard deviation of the distance from the new contour to the consensus.

Differences in mass and volumes derived by new reader contours can be compared with Table 2. The CMR data are available on request from the Cardiac Atlas Project for the purpose of training new readers and benchmarking automated processing methods. Note the consensus is a fusion of a number of different core labs, and does not reflect the practice of any particular lab.

### Limitations

Only fifteen cases of varying pathology were included in the study, due to the time consuming nature of manual contouring. However, the purpose of the study was to provide a resource for assessing automated methods and facilitating training. We therefore chose increased depth of readers from different centres over breadth of subjects. This provides considerable power for analysis of differences [15, 16]. Although resources are becoming available with many hundreds and even thousands of studies [5, 17], these provide manual contours from a small number (typically one or two) of centers. Our study therefore provides a unique resource representing the largest number of expert readers to date.

The SCMR guidelines [11] recommend either inclusion or exclusion of papillary muscles. Our study excluded papillary muscles from the LV mass, but papillary muscles are myocardial tissue, which ideally should be included. The formation of a manual ground truth from many expert readers would be very time consuming, but very valuable for validating automated methods [18, 19]. In the future, more widely automated methods are likely to increase the number of centers quantifying papillary mass. Right ventricular and atria consensus contours would also be useful to establish in the future.

## Conclusion

We have estimated a set of consensus myocardial contours from SSFP CMR short-axis slices from expert readers representing experienced core labs around the world. The consensus contours achieved better agreement in LV mass and volumes than between readers. This consensus dataset is valuable resource for the assessment of new readers, as a basis for multi-center analyses, as well as benchmarking automated methods. The main source of bias was small but consistent differences in contour placement in all areas.

## References

[1] Bluemke DA, Kronmal RA, Lima JA, Liu K, Olson J, Burke GL (2008). The relationship of left ventricular mass and geometry to incident cardiovascular events: the MESA (Multi-Ethnic Study of Atherosclerosis) study. J Am Coll Cardiol.

[2] Gjesdal O, Bluemke DA, Lima JA (2011). Cardiac remodeling at the population level—risk factors, screening, and outcomes. Nat Rev Cardiol.

[3] Grothues F, Smith GC, Moon JC, Bellenger NG, Collins P, Klein HU (2002). Comparison of interstudy reproducibility of cardiovascular magnetic resonance with two-dimensional echocardiography in normal subjects and in patients with heart failure or left ventricular hypertrophy. Am J Cardiol.

[4] Bild DE, Bluemke DA, Burke GL, Detrano R, Diez Roux AV, Folsom AR (2002). Multi-ethnic study of atherosclerosis: objectives and design. Am J Epidemiol.

[5] Petersen SE, Matthews PM, Bamberg F, Bluemke DA, Francis JM, Friedrich MG (2013). Imaging in population science: cardiovascular magnetic resonance in 100,000 participants of UK Biobank—rationale, challenges and approaches. J Cardiovasc Magn Reson.

[6] Karamitsos TD, Hudsmith LE, Selvanayagam JB, Neubauer S, Francis JM (2007). Operator induced variability in left ventricular measurements with cardiovascular magnetic resonance is improved after training. J Cardiovasc Magn Reson.

[7] Pattynama PM, De Roos A, Van der Wall EE, Van Voorthuisen AE (1994). Evaluation of cardiac function with magnetic resonance imaging. Am Heart J.

[8] Bottini PB, Carr AA, Prisant LM, Flickinger FW, Allison JD, Gottdiener JS (1995). Magnetic resonance imaging compared to echocardiography to assess left ventricular mass in the hypertensive patient. Am J Hypertens.

[9] Suinesiaputra A, Cowan BR, Al-Agamy AO, AlAttar MA, Ayache N, Fahmy AS (2014). A collaborative resource to build consensus for automated left ventricular segmentation of cardiac MR images. Medical Image Analysis.

[10] Medrano-Gracia P, Cowan BR, Ambale-Venkatesh B, Bluemke DA, Eng J, Finn JP (2014). Left ventricular shape variation in asymptomatic populations: the multi-ethnic study of atherosclerosis. J. Cardiovasc. Magn. Reson.

[11] Schulz-Menger J, Bluemke DA, Bremerich J, Flamm SD, Fogel MA, Friedrich MG (2013). Standardized image interpretation and post processing in cardiovascular magnetic resonance: Society for Cardiovascular Magnetic Resonance (SCMR) board of trustees task force on standardized post processing. J Cardiovasc Magn Reson.

[12] Warfield SK, Zou KH, Wells WM (2004). Simultaneous truth and performance level estimation (STAPLE): an algorithm for the validation of image segmentation. IEEE Trans Med Imaging.

[13] Dempster AP, Laird NM, Rubin DB (1977). Maximum likelihood from incomplete data via the EM algorithm. J. R. Stat. Soc. Ser. B Methodol.

[14] Medrano-Gracia P, Cowan BR, Bluemke DA, Finn JP, Kadish AH, Lee DC (2013). Atlas-based analysis of cardiac shape and function: correction of regional shape bias due to imaging protocol for population studies. J. Cardiovasc. Magn. Reson.

[15] Abbey CK, Samuelson FW, Gallas BD (2013). Statistical power considerations for a utility endpoint in observer performance studies. Acad Radiol.

[16] Voth M, Attenberger UI, Luckscheiter A, Haneder S, Henzler T, Schoenberg SO (2011). “Number needed to read”—how to facilitate clinical trials in MR-angiography. Eur Radiol.

[17] Fonseca CG, Backhaus M, Bluemke DA, Britten RD, Chung JD, Cowan BR (2011). The Cardiac Atlas Project—an imaging database for computational modeling and statistical atlases of the heart. Bioinformatics.

[18] Papavassiliu T, Kuhl HP, Schroder M, Suselbeck T, Bondarenko O, Bohm CK (2005). Effect of endocardial trabeculae on left ventricular measurements and measurement reproducibility at cardiovascular MR imaging. Radiology.

[19] Vogel-Claussen J, Finn JP, Gomes AS, Hundley GW, Jerosch-Herold M, Pearson G (2006). Left ventricular papillary muscle mass: relationship to left ventricular mass and volumes by magnetic resonance imaging. J Comput Assist Tomogr.

